# Umbilical Hernia

**Published:** 1904-11-19

**Authors:** 


### UMBILICAL HERNIA.

The decision whether to advise a patient having an umbilical hernia to undergo an operation or not is often a troublesome one to make. The reason for this is that in each case there are many important factors to be considered whose value can by no means be exactly calculated. Thus, against operation it may be urged that the patients are usually obese, often have fatty heart, and are bad subjects for surgery. Moreover, the operation is often attended with much difficulty on account of adhesions existing between the sac of the hernia and its contents, and other complications, while there is no certainty of a permanent cure, even if the operation has been well performed and is seemingly satisfactory at the time. In favour of operation are the distress, mental as well as bodily, which the hernia causes, and the possibility of strangulation, an event which, although rare, has additional force because it is attended with a particularly high mortality. In a few cases the reasons for recommending operation may manifestly outweigh the contra-indications or *vice versâ*. Where this happy solution is not possible it will be wise to make no hasty decision. Much may be done by wearing a suitable belt and by careful regulation of the bowels to mitigate the discomforts of the hernia. If, after a sufficient trial of palliative measures, the patient still finds the condition unbearable, or if recurrent signs of inflammation or obstinate constipation occur in an irreducible umbilical enterocele, then operative measures may be suggested after the dangers and drawbacks have been fully explained to the patient or her relatives.

